# Supplementary material for: Resolving the full spectrum of human genome variation using Linked-Reads
Source: Genome Res. 2019 Apr;29(4):635–45. doi: 10.1101/gr.234443.118 (PMC6442396; doi:10.1101/gr.234443.118)
Supplement: Supplemental Material [file supp_gr.234443.118_Supplemental_longranger-2.2.zip › longranger-2.2.tar.bz2!/lr2.2-public/lib/stan/cmdstan/stan/doc/api/html/classstan_1_1mcmc_1_1diag__e__point.html]

Stan: stan::mcmc::diag\_e\_point Class Reference


|  |  |
| --- | --- |
|  | Stan  2.5.0  probability, sampling & optimization |


- Main Page
- Related Pages
- Namespaces
- Classes
- Files

- Class List
- Class Index
- Class Hierarchy
- Class Members

All Classes Namespaces Files Functions Variables Typedefs Enumerations Enumerator Friends Macros Pages

- stan
- mcmc
- diag\_e\_point

Public Member Functions |
Public Attributes |
List of all members

stan::mcmc::diag\_e\_point Class Reference

`#include <diag_e_point.hpp>`

Inheritance diagram for stan::mcmc::diag\_e\_point:

|  |  |
| --- | --- |
| Public Member Functions | |
|  | diag\_e\_point (int n) |
|  | |
|  | diag\_e\_point (const diag\_e\_point &z) |
|  | |
| void | write\_metric (std::ostream \*o) |
|  | |
| Public Member Functions inherited from stan::mcmc::ps\_point | |
|  | ps\_point (int n) |
|  | |
|  | ps\_point (const ps\_point &z) |
|  | |
| ps\_point & | operator= (const ps\_point &z) |
|  | |
| virtual void | get\_param\_names (std::vector< std::string > &model\_names, std::vector< std::string > &names) |
|  | |
| virtual void | get\_params (std::vector< double > &values) |
|  | |

|  |  |
| --- | --- |
| Public Attributes | |
| Eigen::VectorXd | mInv |
|  | |
| Public Attributes inherited from stan::mcmc::ps\_point | |
| Eigen::VectorXd | q |
|  | |
| Eigen::VectorXd | p |
|  | |
| double | V |
|  | |
| Eigen::VectorXd | g |
|  | |

|  |  |
| --- | --- |
| Additional Inherited Members | |
| Static Protected Member Functions inherited from stan::mcmc::ps\_point | |
| template<typename T > | |
| static void | fast\_vector\_copy\_ (Eigen::Matrix< T, Eigen::Dynamic, 1 > &v\_to, const Eigen::Matrix< T, Eigen::Dynamic, 1 > &v\_from) |
|  | |
| template<typename T > | |
| static void | fast\_matrix\_copy\_ (Eigen::Matrix< T, Eigen::Dynamic, Eigen::Dynamic > &v\_to, const Eigen::Matrix< T, Eigen::Dynamic, Eigen::Dynamic > &v\_from) |
|  | |

## Detailed Description

Definition at line 12 of file diag\_e\_point.hpp.

## Constructor & Destructor Documentation

|  |  |  |  |  |  |
| --- | --- | --- | --- | --- | --- |
| stan::mcmc::diag\_e\_point::diag\_e\_point | ( | int | *n* | ) |  |

inline

Definition at line 16 of file diag\_e\_point.hpp.

|  |  |  |  |  |  |
| --- | --- | --- | --- | --- | --- |
| stan::mcmc::diag\_e\_point::diag\_e\_point | ( | const diag\_e\_point & | *z* | ) |  |

inline

Definition at line 22 of file diag\_e\_point.hpp.

## Member Function Documentation

|  |  |  |  |  |  |
| --- | --- | --- | --- | --- | --- |
| void stan::mcmc::diag\_e\_point::write\_metric | ( | std::ostream \* | *o* | ) |  |

inlinevirtual

Reimplemented from stan::mcmc::ps\_point.

Definition at line 26 of file diag\_e\_point.hpp.

## Member Data Documentation

Eigen::VectorXd stan::mcmc::diag\_e\_point::mInv

Definition at line 18 of file diag\_e\_point.hpp.

---

The documentation for this class was generated from the following file:

- src/stan/mcmc/hmc/hamiltonians/diag\_e\_point.hpp


---

[ Stan Home Page ]

*© 2011–2014,
Stan Development Team.*
